# In vitro exposure to very low-level laser modifies expression level of extracellular matrix protein RNAs and mitochondria dynamics in mouse embryonic fibroblasts

**DOI:** 10.1186/s12906-015-0593-8

**Published:** 2015-03-24

**Authors:** Alessandro Giuliani, Luca Lorenzini, Marco Alessandri, Roberta Torricella, Vito Antonio Baldassarro, Luciana Giardino, Laura Calzà

**Affiliations:** Department of Veterinary Medical Sciences, University of Bologna, Via Tolara di Sopra 41/E, 40064 Ozzano Emilia, Bologna, Italy; Health Science and Technologies Interdepartmental Center for Industrial Research (HST-ICIR), University of Bologna, Bologna, Italy; IRET Foundation, Ozzano Emilia, Bologna, Italy

**Keywords:** Ultra Low Level Laser, Neurite elongation, Oxidative stress, Mouse embryonic fibroblast, Matrix protein, Mitochondria dynamic

## Abstract

**Background:**

Low-level lasers working at 633 or 670 nm and emitting extremely low power densities (Ultra Low Level Lasers - ULLL) exert an overall effect of photobiostimulation on cellular metabolism and energy balance. In previous studies, it was demonstrated that ULLL pulsed emission mode regulates neurite elongation *in vitro* and exerts protective action against oxidative stress.

**Methods:**

In this study the action of ULLL supplied in both pulsed and continuous mode vs continuous LLL on fibroblast cultures (Mouse Embryonic Fibroblast-MEF) was tested, focusing on mitochondria network and the expression level of mRNA encoding for proteins involved in the cell-matrix adhesion.

**Results:**

It was shown that ULLL at 670 nm, at extremely low average power output (0.21 mW/ cm^2^) and dose (4.3 mJ/ cm^2^), when dispensed in pulsed mode (PW), but not in continuous mode (CW) supplied at both at very low (0.21 mW/cm^2^) and low levels (500 mW/cm^2^), modifies mitochondria network dynamics, as well as expression level of mRNA encoding for selective matrix proteins in MEF, e.g. collagen type 1α1 and integrin α5.

**Conclusions:**

We suggest that pulsatility, but not energy density, is crucial in regulating expression level of collagen I and integrin α5 in fibroblasts by ULLL.

## Background

The use of Low Density/Power lasers in physical medicine (Low Level Laser Therapy- LLLT) has become a consolidated practice in recent decades. Part of the wide range of therapeutic indications have been validated by meta-analysis studies, such as tendon injuries [1], wound healing [2], pain [3-6], nerve regeneration [7-9]. The main physical characteristics of LLLT are the wavelength range from far-red to near infrared (600–1064 nm) and power range from 0.001 to 5 W/cm^2^ with an application period of a few seconds to several minutes [10]. Safety profile [11] and dosage recommendations are available from the World Association for Laser Therapy [12].

Cellular, subcellular and molecular mechanisms underlying the medical effects of LLLT are under intensive investigation, and agreement is emerging that mitochondria are the principal photoacceptors present inside cells [13,14]. Moreover, a number of papers reporting *in vitro* and *in vivo* studies indicate that the intracellular pathways affected by LLL stimulation include oxidative stress-related pathways [15], PI3-K/Akt signalling cascade and nuclear receptors [15], NFkB [16] and others [17,18]. These pathways are under active investigation to elucidate the possible mechanism of the therapeutic actions of LLLT, also pursuing the objectives of Evidence Based Medicine.

More recently, a new class of low-level lasers working at 633 or 670 nm and emitting extremely low power densities (about 0.15 mW/cm^2^) has been introduced. These Ultra Low Level Lasers (ULLL) working in pulsed or continuous emission mode are capable of eliciting significant biological effects, possibly via photostimulation [10]. ULLL therapeutic effectiveness on human and animal models has been suggested in osteoarthritis [19], balance disorder when applied on acupoints [20], acute and chronic joint inflammation [21,22], thermal hyperalgesia in rat [23], orthodontic applications [23], cosmetic medicine [24], wound healing [25], etc.

In this study the action of ULLL delivered in both pulsed and continuous mode vs continuous LLL was tested on fibroblast cultures (Mouse Embryonic Fibroblast-MEF), focusing on the expression level of proteins involved in the cell-matrix adhesion. Sub-cellular effects of ULLL pulsed emission mode has been demonstrated *in vitro* on neurite elongation [26], on cell body shape [27], as protective action from oxidative stress [10,26]. As the fibroblasts are the most abundant cellular component of the subcutaneous tissue, these cells are primarily and directly affected by the laser beam during laser therapy, in conditions in which the subcutaneous tissue is the target for (as for wound healing and non invasive body contouring) or is incidentally invested (as for orthopaedic pathologies) by ULLL therapy.

## Methods

### MEF cell culture and exposure system

**Figure 1:**
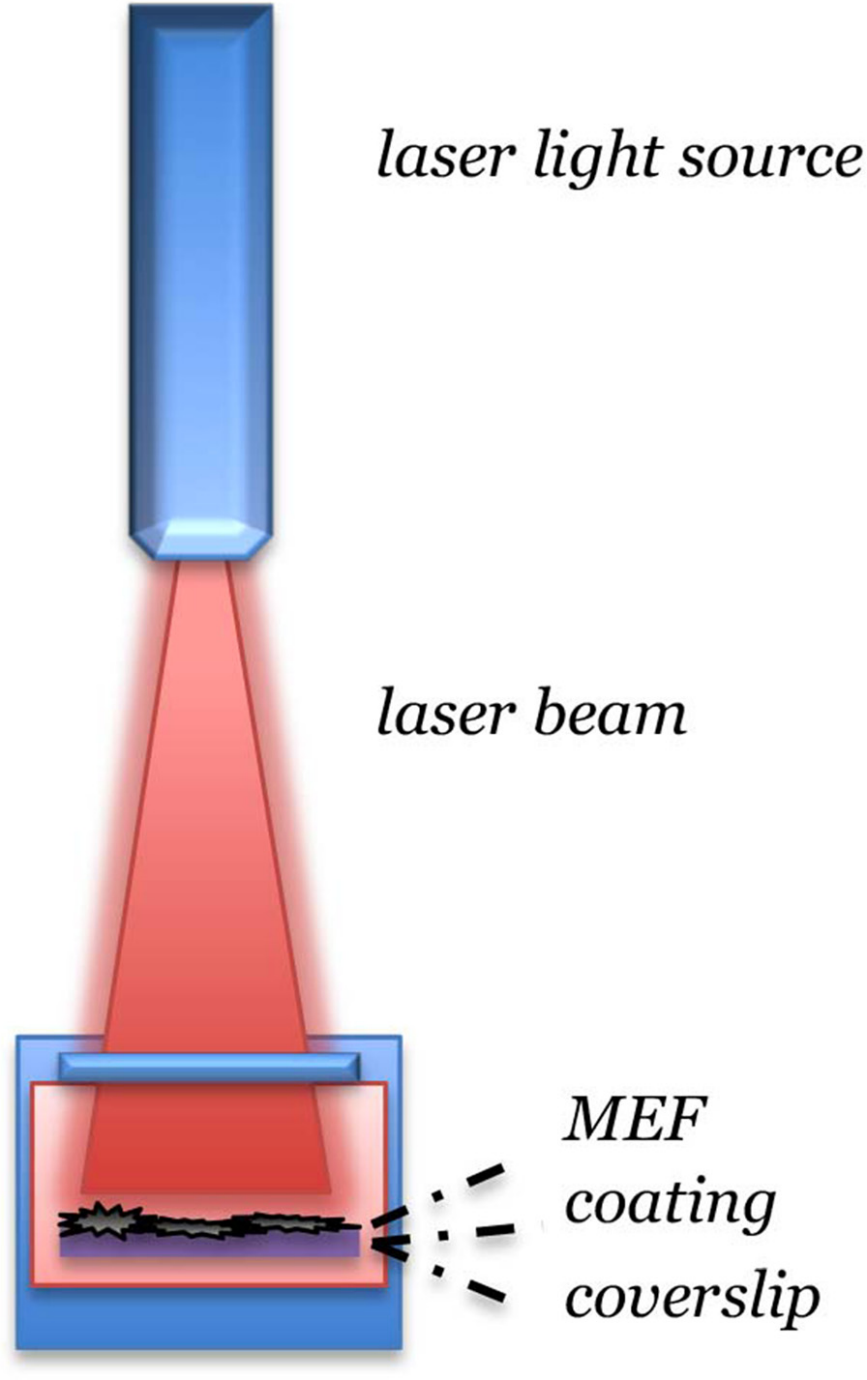
**Exposure system on MEF culture used in the study.**

Mouse Embryonic Fibroblast*s* (MEFs) were prepared at day 12.5-14.5 as described [28]. Cells were seeded at a density of 1.6×10^5^ and 5×10^3^cells/well in a 24 multi-well plate with glass coverslips 2D-Cultrex® BME coated for mRNA extraction and ImmunoCytoChemistry (ICC), respectively. MEF were maintained in a humidified atmosphere at 37°C, 5% CO_2_ in a medium consisting of DMEM (Dulbecco's Modified Eagle's Medium) (GIBCO), 10% FBS, 1% MEM/NEAA (Minimum essential medium/nonessential amino acids) (GIBCO), 100 IU/ml Penicillin/100 μg/ml Streptomycin (GIBCO). The Biolite® exposure system (Figure 1) consisted of a SANYO DL3149-055A diode with wavelength = 670 ± 10 nm; peak power = 3 mW. The following emission modes were used: CW = Continuous Wave; PW = Double Pulsed Square Wave (Biolite® int. patent). A CW Reference LLL class laser was also included. Emission parameters of all exposure conditions are listed in Table 1. These power/energy levels lie well outside the limits set by the Arndt-Shulz law for photostimulation [29] and have been discussed in a specific report [10]. The “reference” laser probe (CW Ref) consists of a Continuous Wave Diode with wavelength =650 nm, and peak power = 50 mW.

Twenty-four h after seeding, cells were exposed for 20” once a day for 3 consecutive days. For immunocytochemistry experiments, in order to investigate mitochondrial network dynamic immediately after laser, cells were fixed after the last laser irradiation. In order to investigate the effect of laser irradiation on mRNA expression level cells were processed 2 h after last laser irradiation.

### Immunocytochemistry, imaging and image analysis

Indirect immunofluorescence (IF) procedures were used for cytochrome C (Cyt-C) detection. Cells were washed in PBS and fixed in 4% paraformaldehyde in 0.1 M Sørensen phosphate buffer for 20 min at room temperature (RT). Cells were then blocked with 5% Donkey Normal Serum (Sigma-Aldrich) in 0.3% PBS/Triton-X 100 (Merck, Darmstadt, Germany) for 1 h at room temperature, then incubated overnight at 4°C in humid atmosphere with the primary antibody (sheep anti-Cyt-C: 1:800; Sigma-Aldrich) diluted in blocking solution. After rinsing in PBS (2×10min), cells were incubated with fluorochrome-conjugated secondary antisera (DyLight 488 Donkey Anti-Goat 1: 500; Jackson ImmunoResearch Laboratory) diluted in 0.3% PBS/Triton-X 100 for 30 min at 37°C. For nuclear staining, cells were first washed in PBS then incubated 15 min in PBS containing 1 μg/ml Hoechst 33258, 0.2% Triton-X 100. After rinsing in PBS cells were mounted in 0.1% glycerol/1,4-phenylendiamine (Sigma-Aldrich). Negative controls were performed by primary antibody omission.

**Figure 2:**
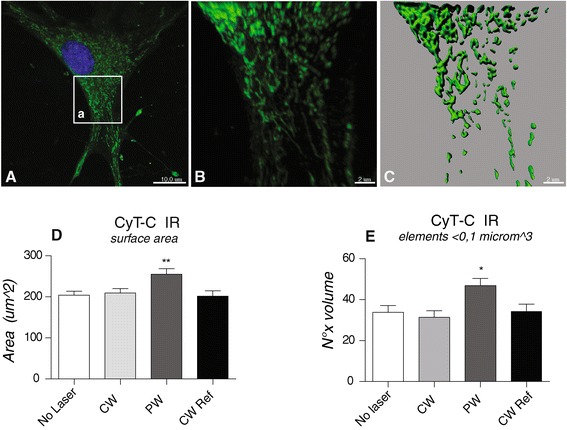
**Mitochondria dynamic analysis in MEF under the different experimental conditions. A**. Confocal image of Cyt-C-IR fibroblast. The square in a) indicates the sampled area. **B-C**. Image processing: **B)** Background subtraction and gaussian smoothing filter were applied using Imaris software (BitPlane); **C)** Mitochondrial isosurface reconstruction was obtained with 3D analysis software Imaris. **D**. Quantitative analysis of total Cyt-C-IR isosurface area. There are no differences between unexposed cells and cells exposed to CW mode, while there is an increase in the values of the total isosurfaces of the cells exposed to PW. *Statistical analysis:* One way ANOVA, Dunnett’s multiple comparison test **p < 0.01. **E**. Number of CyT-C IR elements with a volume included between 0.04 and 0.1micron^3. There is an increase in the number of mitochondria in cells exposed to PW Mode. *Statistical analysis:* one way ANOVA, Dunnett’s multiple comparison test *p < 0.05.

Analysis was carried out on adherent fibroblasts presenting lamellipodia and filopodia. Fifty cells for each treatment group were analyzed by Nikon Ti-E fluorescence microscope coupled with A1R confocal system (Nikon). A multi-Ar (457/488/514) laser with exciting wavelengths for DyLight 488 was used. Images were acquired by oil-immersion 60x objective (NA 1.4) and a 6x zoom factor, using Nis-Elements AR 3.2 software. Image resolution was 1024×1024 pixels and optical sections for confocal stacks were 0.125 micrometers. The image frame for mitochondrial network analysis was positioned in the cytoplasmic area of the cell so that at least one side of the frame was in contact with the nucleus. The mitochondria network was sampled as volume surface of Cyt-C-IR objects, created and analyzed on confocal stacks using the 3D analysis software Imaris (Bitplane AB, Saint Paul, MN, USA). For isosurface creation and measure, images were processed by background subtraction and gaussian smoothing filter, then normalized according to the sampled volume (Figure 2). To distinguish mitochondria from residual background, objects with a volume of less than 0.04 μm^3^ were excluded from the analysis, while objects with a volume ranging from 0.04 to 0.1 μm^3^ and were considered as single mitochondria [29].

### RNA extraction, retrotranscription and semi-quantitative real time PCR

Total RNA was extracted using Mini RNAasy Kit (Qiagen, Milan, Italy) and following the manufacturer’s specifications. RNAs were subjected to DNase treatment (1 U/μl, 1x DNase buffer, 2 U/μl ribonuclease inhibitor at 37°C for 30 min) (Fermentas, Life Sciences, Italy) and mRNAs were retrotranscribed with the enzyme M-Moloney murine leukemia virus reverse transcriptase (M-MuLV-RT, 10Uμl) (Fermentas) in the presence of 1x first strand buffer, 1 mM d(NTP)s (Fermentas), 25 ng/μl Oligo (dT)_18_ primer (Fermentas), incubating at 42°C for 60 min.

The mRNA expression analyses were performed using a Real Time PCR (Mx3005P QPCR System, Stratagene, La Jolla, CA, USA) equipped with a FAM™/SYBR® Green I filter (492 nm excitation-516 nm emission). The chemistry chosen to perform these PCR experiments was based on SYBR Green I fluorescent detection and each reaction mix consisted of 10 ng of template cDNA, 1x Maxima™ SYBR Green/ROX qPCR Master Mix (Fermentas) and 0.4 μM of both primers (sense and antisense). Specific primers for each gene of interest are summarized in Table 2; among these, GAPDH was considered as housekeeping gene. All primers were obtained from IDT (Coralville, IA, USA). PCR started with 1 cycle at 95°C for 10 min, followed by 40 cycles with specific conditions for each primer, as shown in Table 2; at the end of amplification cycles the dissociation curve was constructed following a procedure consisting of first incubating sample at 95°C for 1 min to denature the PCR-amplified products, then ramping temperature down to 55°C and finally increasing temperature from 55°C to 95°C at the rate of 0.2°C/s, continuously collecting fluorescence intensity over the temperature ramp. The specificity of the amplified product was controlled by the presence of a single peak at the expected melting temperature. Random amplified products were resolved by electrophoresis in a 2.0% agarose gel stained with ethidium bromide, in order to check the specificity of the PCR reaction. This was confirmed by the presence of a single band of the expected size. A 100 bp DNA ladder (Fermentas) was used as DNA marker. The semi-quantitative analysis of gene expression was performed on the values of the threshold cycle (Ct) obtained for each sample, considering GAPDH as housekeeping gene. Samples were always processed in duplicate. The relative gene expression was calculated with the formula 2^(−△△Ct)^, using a defined group as reference (2^(−△△Ct)^ = 1).

**Table 1:** **Laser emission parameters**

**Parameter**	**Unity**	**Laser emission**		
		**CW**	**PW**	**CW Ref**
**Wavelenght**	nm	670	670	650
**Modulation**	--	none	Pulsed	none
			100 Hz – 1%	
			+1 Hz 50%	
**Spot size**	cm^2^	0.07	0.07	0.10
**Peak power**	mW	3	3	50
**Average power**	mW	3	0.015	50
**Avg fluence**	mW/cm2	42	0.21	500
**Emission time**	sec	20	20	20
**Energy**	mJ	60	0.3	1,000
**Energy density**	mJ/cm2	857	4.3	7,692

**Table 2:** **Primers and conditions used for real time PCR reactions**

**Gene**	***Acc. N°***	***Sequences (x-y)***	**Conditions**
*GAPDH*	M17701	5-GGCAAGTTCAATGGCACAGTCAAG-3	95°C 30s
		5-ACATACTCAGCACCAGCATCACC-3	60°C 30s
			40 cycle
*Laminin α1*	NM_008480.2	5-GGGATGAAGAAGCAAAGCAACT-3	95°C 30s
		5-CTCCTTTGCAACACTGCTGTC-3	60°C 45 s
			40 cycle
*Cadherin 1*	NM_009864.2	5-CGACCGGAAGTGACTCGAAA-3	95°C 30s
		5-AACCACTGCCCTCGTAATCG-3	60°C 45 s
			40 cycle
*Integrin α5*	NM_010577.3	5-CTCTGTGGCTGTGGGTGAAT-3	95°C 30s
		5-CGAAGTAGGAGGCCATCTGTT-3	60°C 45 s
			40 cycle
*Collagen type I α1*	NM_007742.3	5-TCAGCTTTGTGGACCTCCG-3	95°C 30s
		5-GGACCCTTAGGCCATTGTGT-3	60°C 45 s
			40 cycle

### Statistical analysis

Statistical testing was performed with ANOVA followed by Dunnett’s multiple comparisons test. A probability level of p < 0.05 was considered to be statistically significant.

## Results

In these experiments, the fibroblasts were grown on Cultrex®-coated slides. Cultrex® is a soluble form of basement membrane, the continuous sheets of specialized extracellular matrix that form an interface between cells and their adjacent stroma. The Cultrex® composition includes laminin, collagen IV, entactin, and heparin sulfate proteoglycan. Thus, this culturing condition provides an in vitro adhesion surface close to the physiological microenvironment of fibroblasts.

In order to confirm that the ULLL irradiation used in this study impacts on mitochondria, e.g. the well-established photoaceptor organell, in a first set of experiments the mitochondria net based on Cyt-C immunoreactive signal were analyzed. An increase was found in the total isosurfaces in cells exposed to PW compared to both CW and CW Ref mode (p = 0.0061) (Figure 2). This suggests that the number of isolated mitochondria increases under this irradiation condition, as confirmed by the count of elements having volume lying between 0.04 and 0.1 μm^3^ (p = 0.0195).

**Figure 3:**
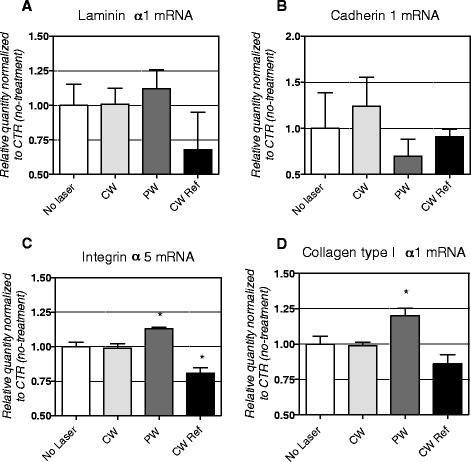
**PCR analysis of the expression level of mRNA encoding for extracellular matrix and adhesion proteins.** The exposure to CW, ref CW and PW laser modulations had no effect on the expression of glycoprotein laminin α1 **(A)** and cadherin 1 **(B)**. PW Mode irradiation increased integrin α5 mRNA (**C**, *Statistical analysis:* one way ANOVA*, Dunnett’s multiple comparison test* * p < 0.05), while the ref CW decrease its expression. (*Statistical analysis:* one way ANOVA*, Dunnett’s multiple comparison test* * p < 0.05). PW also increased collagen type1α1 mRNA (**D**, *Statistical analysis:* one way ANOVA*, Dunnett’s multiple comparison test* * p < 0.05).

In view of the impact of therapeutic low-level laser irradiation on the subcutaneous tissue and resident cells, in a second set of experiments the expression level of mRNA encoding for extracellular matrix and adhesion proteins in MEF was analysed (Figure 3). PW induces an increase in integrin α5 (p = 0.0192) and collagen type1α1 (p = 0.0263) mRNAs expression level, while it had no effect on laminin α1 and cadherin 1.

## Discussion

The present study shows that ULLL at 670 nm, at extremely low average power output (0.21 mW/cm^2^) and dose (4,3 mJ/cm^2^), when dispensed in pulsed mode (PW), but not in continuous mode (CW) delivered both at very low (0.21 mW/cm^2^) and at low levels (500 mW/cm^2^), modifies mitochondria network dynamics, and expression level of mRNA encoding for selective matrix proteins in MEF, e.g. collagen type 1α1 and integrin α5. Notably, continuous wave light, which is the gold standard for all LLLT applications [30], is not effective on these biological targets. It was thus confirmed that pulsed photostimulation rather than dose is a key characteristic of biologically effective ULLL therapy.

Pulsed light could interfere with some fundamental frequencies (2.5-10.0 Hz range) and/or processes that exist in biological systems, including photodissociation in cytochrome c oxidase in mitochondria [31,32]. Mitochondria are in fact recognized as the principal intracellular target of red and near-infrared light [33]. The wavelength used in this study was 670 nm, one of the four suggested "active zones" (peak positions between 667.5 and 683.7 nm) for LLLT [33]. This corresponds to the absorption spectrum of oxidized cytochrome c oxidase [33], leading to improvement in electron transport, increased mitochondrial membrane potential (MMP), and greater ATP production [15]. Wavelengths lying between 650 and 680 nm also induced a significant up-regulation of gene expression in pathways involved in mitochondrial energy production and antioxidant cellular protection [34,35]. In previous studies, it was demonstrated that this ULLL protects cells from mitochondria-mediated cell death [26]. It is reported herein that mitochondria dynamics is influenced by pulsed photostimulation, and this is a quite significant result. Indeed, while this is a known phenomenon in plant cells [36], the light-dependent redistribution of mitochondria along actin or microtubule has been much less explored in mammalian cells. The analysis was performed on confocal images using a 3D-voxel image analysis software, and the mitochondria volume detected by this analysis was according to Kaasik et al. [30]. When observed in fibroblast cultures, mitochondria morphology is found to be far from static, changing continually through the phenomena of fission and fusion within minutes. The coordination between fusion and fission contributes to the response of mammalian cells to stress, and mitochondrial bioenergetics seems to be strongly dependent on mitochondrial morphology [37]. It may thus be suggested that also the effect on mitochondria dynamics is part of the overall effect of photobiostimulation on cellular metabolism and energy balance.

In view of the therapeutic application of LLL therapy to wound healing [25] and cosmetic tissue restructuring [38], the effect of ULLL irradiation on expression profile of mRNA encoding for proteins involved in extracellular matrix composition and cell-matrix interaction was then explored. As exploratory molecular targets, and in view of data previously published by our lab [27], collagen type 1α1, integrin α5, laminin α1, cadherins were investigated in irradiated fibroblasts. Collagen type 1α1 is produced from the MEF and represents 90% of total collagen in most connective tissues; integrin α5 is responsible for binding macromolecules to the matrix and is involved in angiogenesis; laminin α1 is the glycoprotein responsible for the binding to the extracellular matrix; cadherins are transmembrane proteins that mediate cell-cell adhesion in animals. By using a real-time PCR, it was shown that the expression level of mRNA encoding for collagen type 1α1 and integrin α5, but not laminin α1 and cadherin 1, is upregulated by pulsed, but not by continuous ULLL irradiation.

It is well established that collagen production by fibroblasts is affected by LLLT in different body districts, such as cartilage [39], muscle [40], mucosa [41], large vessel walls [42], when used in the red-near infrared wavelength, at an energy density of around 1-5 J/cm^2^. Moreover, also gene expression profiles of genes belonging to different categories, including cytoskeleton and cell-cell interaction, is altered upon similar irradiation in human fibroblasts [43]. Expression level of mRNAs and/or proteins belonging to the large integrine family are also affected by LLL irradiation, as found in bone [44] and in human adipose derived stem cells [45]. In our study, a comparable wavelength (670 nm) was used, but at a dose about a thousand times lower (4.3 mJ/cm^2^) than in the above-mentioned studies.

## Conclusions

These date are part of a large study aimed to highlight the affect of photostimulation by LLLT on different cellular functions. From these results we can conclude that pulsatility, but not energy density, is crucial in regulating the expression level of collagen I and integrin α5 in fibroblasts. This regulation could be also part of the reshaping effect of ULLL irradiation in fibroblasts [26] and of HeLa cell attachment in vitro [46,47]. However, dose–response experiments are still necessary, such as the analysis of the effect of different wavelength, to better characterize the biological impact of photostimulation by LLLT.
